# BOMB trial: First results of stereotactic radiotherapy to primary breast tumor in metastatic breast cancer patients

**DOI:** 10.3389/fonc.2023.1062355

**Published:** 2023-03-17

**Authors:** Edy Ippolito, Sonia Silipigni, Francesco Pantano, Paolo Matteucci, Sofia Carrafiello, Maristella Marrocco, Rita Alaimo, Vincenzo Palumbo, Michele Fiore, Paolo Orsaria, Rolando Maria D’Angelillo, Vittorio Altomare, Giuseppe Tonini, Sara Ramella

**Affiliations:** ^1^ Department of Radiation Oncology (Medicine and Surgery), Università Campus Bio-Medico di Roma, Rome, Italy; ^2^ Radiation Oncology, Fondazione Policlinico Universitario Campus Bio-Medico, Rome, Italy; ^3^ Department of Medical Oncology (Medicine and Surgery), Università Campus Bio-Medico di Roma, Rome, Italy; ^4^ Medical Oncology, Fondazione Policlinico Universitario Campus Bio-Medico, Rome, Italy; ^5^ Department of Radiation Oncology, Azienda Sanitaria Locale (ASL) Frosinone, Hospital of Sora, Sora, Italy; ^6^ Department of Breast Surgery (Medicine and Surgery), Università Campus Bio-Medico di Roma, Rome, Italy; ^7^ Breast Surgery, Fondazione Policlinico Universitario Campus Bio-Medico, Rome, Italy; ^8^ Radiation Oncology, Department of Biomedicine and Prevention University of Rome “Tor Vergata”, Rome, Italy

**Keywords:** stereotactic radiation (SBRT), breast cancer, metastatic patients, safety, outcome

## Abstract

**Aim:**

A prospective dose escalation trial was developed to evaluate the maximum tolerated dose of stereotactic body radiotherapy (SABRT) to primary breast cancer in stage IV disease. The aim of the present report was to describe safety and outcome of the first dose level cohort of patients.

**Material and methods:**

Patients with histologically confirmed diagnosis of invasive breast carcinoma (biological immuno-histochemical profile: luminal and/or HER2 positive) and distant metastatic disease not progressing after 6 months of systemic therapy with a tumor CT or 5FDG-PET detectable were deemed eligible. The starting dose was 40 Gy in 5 fractions (level 1) because this dose proved to be safe in previous dose-escalation trial on adjuvant stereotactic body radiotherapy. The maximum dose level was chosen as 45 Gy in 5 fractions. Dose limiting toxicity was any grade 3 or worse toxicity according to CTCAE v.4. Time-to-event Keyboard (TITE-Keyboard) design (Lin and Yuan, Biostatistics 2019) was used to find the maximum tolerated dose (MTD). MTD was the dose of radiotherapy associated with a ≤ 20% rate pre-specified treatment-related dose-limiting toxicity (DLT).

**Results:**

To date 10 patients have been treated at the starting dose level. Median age was 80 years (range 50-89). 7 patients had a luminal disease, while 3 patients had an HER2 positive disease. No patient suspended ongoing systemic treatment. No protocol defined DLTs were observed. Grade 2 skin toxicity occurred in 4 patients with diseases located close to or involving the skin. Median follow-up was 13 months and all 10 patients were evaluable for response: 5 achieved a complete response, 3 achieved a partial response and 2 showed a stable disease, all with a clinical benefit (resolution of skin retraction, bleeding and pain). The mean reduction in the sum of the largest diameters of target lesions was of 61.4% (DS=17.0%).

**Conclusions:**

SABR to primary breast cancer seems feasible and is associated with symptoms reduction. Continued accrual to this study is needed to confirm the safety and assess the MTD.

**Clinical trial registration:**

ClinicalTrials.gov, identifier NCT05229575.

## Introduction

Approximately 5-10% of women present *de novo* metastatic breast cancer (MBC). This is still an incurable disease even if in the last decades several advances in the treatment of MBC patients have significantly prolonged survival over time (1).

Loco-regional treatment in these patients is highly debatable and its role is not yet established.

It appears that preclinical studies could justify the addition of local treatment to systemic therapy. In fact, the primary breast tumor can be a reservoir of cancer stem cells (2), and can secrete growth factors involved in implantation and growth of metastatic sites (3). Moreover, studies in animal models suggest that resection of the primary breast tumor in mice can restore the immuno-competence of the host (4).

On the other hand, clinical trials results are controversial. A Cochrane systematic review on this topic was recently published, including two randomized studies. Breast surgery showed improved local progression-free survival (HR 0.22, 95% CI 0.08 to 0.57; 2 studies; 607 women), but worsened distant progression-free survival (HR 1.42, 95% CI 1.08 to 1.86) in one study. The authors concluded that with the available data, the decision to perform breast surgery on these women should be individualized and shared between the physician and the patient, carefully considering the potential risks, benefits, and costs (5). More recently, results from EA2108 randomized trials were published showing that early loco-regional therapy for the primary site was associated with improved loco-regional control, but did not improve survival (6).

However, even if current data does not support loco-regional treatment, some of the results of randomized trials, together with the findings of several retrospective studies, suggest that there could be a subset of patients presenting long progression-free survival who might benefit from radical loco-regional treatment. Moreover, treatment of primary breast cancer may provide clinical benefits eventually reducing related symptoms such as pain, bleeding, and skin retraction (7).

There is not much data available on radiotherapy alone for the primary tumor in metastatic breast cancer patients.

A French study retrospectively evaluated the impact of loco-regional treatment (mostly radiotherapy) in a well-selected stage IV breast cancer patients group (33% single metastatic site, 49% without visceral metastases). With a median follow-up of 6.5 years, loco-regional treatment obtained a durable local control of 85%. Particularly, radiotherapy alone compared with surgery followed by radiotherapy provided similar outcomes in terms of overall survival and metastatic-free survival after adjustments for prognostic factors (8).

Stereotactic ablative body radiotherapy (SABR) is a safe and effective treatment modality in several tumor sites, including lung, brain and liver (9, 10),. In breast cancer treatment, SABR has been mainly studied in the neoadjuvant and adjuvant settings (11–13). To date, SABR to intact primary tumors without subsequent surgery has not being investigated, even in palliation setting in metastatic patients. However, SABR has many potential advantages such as the radio-biological advantage of a short and highly effective schedule, the possibility of preventing lesions from becoming symptomatic, as well as the possibility of not interrupting systemic therapy.

Taking into consideration the aforementioned, we started an ongoing phase I trial (see ClinicalTrials.gov Identifier: NCT05229575) to assess the maximum tolerated dose of SABR to primary breast cancer tumors in stage IV patients. The present report aimed to describe the outcome of the first dose-level patients.

## Material and methods

All women 18 years of age or older presenting with a histologically confirmed diagnosis of invasive breast carcinoma (biological immuno-histochemical profile: luminal and/or HER2 positive) and stage IV disease not experiencing extra-mammary disease progression after 6 months of systemic therapy, primary unifocal tumor < 5 cm detectable at either CT or FDG PET-CT scans, were eligible for inclusion. All patients recommended for surgery were excluded. Our institutional ethics committee approved the trial, and informed consent was obtained from all participants.

Simulation and treatment were performed with the patient in the supine position using a breast board (Civco Medical Solutions, Orange City, IA, U.S.A.). The gross tumor volume (GTV) was defined as the primary breast lesion seen on CT scan or 5-FDG PET-CT scan. The clinical target volume was defined as the GTV plus a 3 mm margin. The planning target volume (PTV) was defined as the clinical target volume plus an internal margin as defined by 4a DCT scan or plus a 3 mm margin if breath hold was employed. Critical structures included the heart, lungs, skin, both breasts, and the chest wall.

The dose was prescribed at the edge of the PTV. To be approved, the 80% isodose line prescription needed to encompass 100% of the PTV volume.

The trial’s primary endpoint was to establish the maximum tolerated dose (MTD) of stereotactic body radiotherapy for breast primary tumors. The MTD was considered as the dose of radiotherapy associated with a ≤ 20% rate of e pre-specified treatment-related dose-limiting toxicity (DLT) occurring within 6 months from the start of treatment. DLT was defined as any grade 3 or worse toxicity according to CTCAE v.4.02. If no DLT occurs, the trial aims to recruit a total of 30 patients. The goal of the present interim analysis was to assess with a minimum follow-up of 6 months toxicity experienced by the first dose level cohort of patients. The radiological response was also assessed.

Patients received 5 fractions of radiation, on a 2-day basis. The starting dose level was 40 Gy in 5 fractions (level 1) as this dose was deemed safe in a previous dose-escalation trial on adjuvant stereotactic body radiotherapy (13), which corresponds to a 2 Gy equivalent dose of 76 Gy (biologically equivalent dose- BED 110 Gy, alpha/beta 4.6 Gy). The highest dose level was set at 45 Gy in 5 fractions, equivalent to 93 Gy delivered in 2 Gy fractions (BED4.6Gy= 133 Gy). According to older studies on radiotherapy as the definitive treatment in breast cancer (14, 15), this dose should be associated wa ith very low local recurrence risk rate (<15%)

Ipsilateral breast and axillary ultrasonography and a chest CT scan were performed 45 days after rt and thereafter according to the physician’s prerogative. The radiologic response was evaluated according to the Response Evaluation Criteria in Solid Tumors. NCI CTCAE v 4.02 scale for radiation dermatitis, breast pain, breast infection, breast asymmetry, fibrosis, skin atrophy, rib fracture, or chest wall pain was used. Photographic documentation was carried out at each clinical evaluation.

## Results

Between August 2019 and June 2021, 10 patients were enrolled and treated at the starting dose level. The median age was 80 years (range 50-89). Seven patients had a luminal disease, while 3 patients had HER2-positive disease. No patient suspended ongoing systemic treatment (see **Table 1** for details), median tumor diameter, measured on pre-treatment ultrasonography was 20 mm (range: 10 mm–46 mm). Before starting radiotherapy 3 patients showed skin retraction, 1 patient skin ulceration and bleeding and 2 patients presented breast pain. All patients received radiotherapy course as planned. Planning data is listed in **Table 2**.

**Table 1:** Patients’ clinical characteristics.

Patient	Age	Laterality (right/left)	Molecular subtype	Concurrent systemic treatment	Site of Metastatic disease
1	80	left	HER2 Luminal	Capecitabine/Lapatinib	bone
2	53	left	Luminal	Palbociclib + Aromatase inhibitors	bone, liver, lung
3	89	right	Luminal	Aromatase inhibitors	lung
4	80	left	Luminal	Palbociclib + Aromatase inhibitors	liver
5	84	left	Luminal	Aromatase inhibitors	lung
6	85	left	Luminal	Aromatase inhibitors	lung
7	56	right	HER2 Luminal	Capecitabine/Lapatinib	brain, liver
8	82	left	Luminal	Aromatase inhibitors	lung
9	50	right	Luminal	Ribociclib + Aromatase inhibitors	bone, lung
10	76	left	HER2	Trastuzumab/pertuzumab dual blockade	bone

**Table 2:** Planning data and toxicity description.

Patient	GTV (cc)	PTV (cc)	Uninvolved ipsilateral breast (dose to 50% of volume)	Maximum point dose contralateral breast (Gy)	Skin dose (10 cc)	Chest wall dose (10 cc)	Lung D10% (Gy)	Heart Dmean (Gy)	Toxicity (CTCAE v 4.02)
1	16.9	38.8	1.2	0.3	1.7	16.8	4.0	0.4	–
2	12.4	24.8	3.1	1.2	2.0	21.7	5.1	1.5	–
3	3.30	24.2	2.5	0.7	26.7	11.2	3.0	0.4	Grade 2 radiation dermatitis Grade 1 hyperpigmentation
4	27.5	75.2	1.9	1.1	9.9	29.8	5.3	0.2	Grade 1 breast induration, Grade 1 breast pain
5	15.4	33.2	4.5	1.2	40.3	9.6	1.5	0.8	Grade 2 radiation dermatitis Grade 1 hyperpigmentation
6	14.7	44.8	1.2	13.8	27.6	37.4	0.2	1.5	Grade 2 radiation dermatitis Grade 1 hyperpigmentation
7	18.5	32.3	2.5	1.5	22.6	5.8	2.4	0.7	Grade 1 radiation dermatitis Grade 2 hyperpigmentation
8	12.8	61.2	1.1	9.4	13.9	29.3	4.5	0.3	Grade 1 radiation dermatitis
9	16.2	46.8	1.2	2.4	18.5	13.0	5.6	1.5	Grade 1 lung fibrosis
10	15.7	39.3	1.3	3.0	22.6	29.1	7.0	0.7	Grade 2 radiation dermatitis, Grade 1 breast induration Grade 2 hyperpigmentation

No protocol-defined DLTs were observed. There were 6 acute grade 1 toxicity events (breast pain, hyperpigmentation, radiation dermatitis) and 4 acute grade 2 skin dermatitis. All of the latter occurred in 4 patients with diseases located next to the skin and showing clinical retraction. In these patients, the medium maximum dose delivered to 10 cc of skin was 30.1 Gy. In all of these patients, bolus was employed. To date, no grade ≥2 late toxicity events occurred; 1 patient developed grade 1 lung fibrosis, and 2 patients G1 breast induration. See **Table 2** for details.

The median follow-up of the entire cohort was 13 months (7-24 months). To date, 7 patients are alive (2 died due to brain progression and 1 for cardiovascular disease). The median largest tumor diameter, measured on post-treatment ultrasonography was 10.5 mm (range: 5 mm–41 mm). The mean reduction in the sum of the largest diameters of target lesions was 61.4% (DS=17.0%).

At 1 year, the local control was 100%. All 10 patients were evaluable for response: 5 achieved a complete response, 3 achieved a partial response and 2 showed stable disease. The 2 patients showing stable disease presented with skin retraction. In both patients, skin retraction disappeared after treatment.

Overall, patients achieved a resolution of pre-radiotherapy clinical symptoms (skin retraction, bleeding and pain).

**Figure 1:**
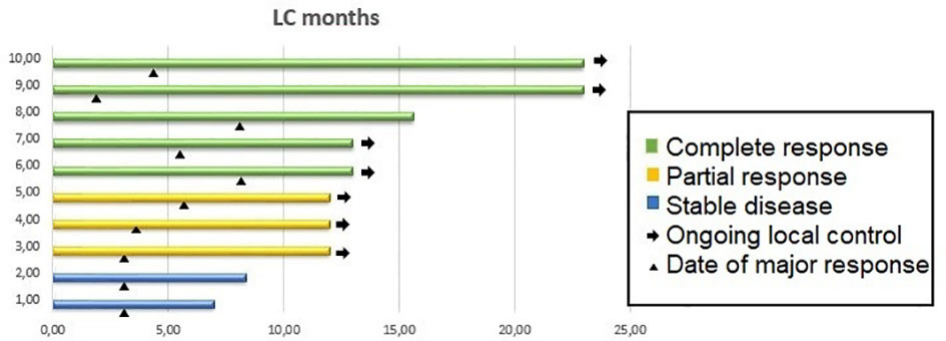
Local control duration, response, and date of major response.

**Figure 1** shows local control duration, response, and date of major response.

## Discussion

In this ongoing pilot study, we are looking to find the MTD of stereotactic body radiotherapy for luminal and/or HER 2 positive breast primary tumors in stage IV disease patients, delivered during systemic treatment. To the best of our knowledge, no study has investigated the use of SABRT in this setting, consequently, we needed to undergo a dose-finding study.

Historical series on conventionally fractionated radiotherapy alone as definitive breast cancer treatment demonstrated tumor dose as being significantly related to local disease control (14). Arriagada et al. evaluated 463 breast cancer patients treated with radiotherapy alone at the Princess Margaret Hospital and at the Institut Gustave-Roussy. Analysis of local control showed that a radiation dose increase of 15 Gy can lower the relative risk of tumor or lymph node recurrence twofold (14). Van Limbergen et al. (15), reviewing data on 221 breast cancer patients treated with radiotherapy alone reported that a 10 Gy higher dose for T1 tumors and 35 Gy higher dose for T2 tumors was needed to provide local control rates similar to a combination of surgery and radiation. The rate of local control ranged from 70 to 80% when doses higher than 70 Gy were applied. However, higher doses were associated with poorer cosmetic results, with only 15% of patients who received more than 80 Gy having good cosmetic results (15).

More recently, Shibamoto et al, reported the results of a study investigating a curative radiotherapy treatment for patients with primary operable breast cancer who refused surgery. Radiotherapy doses delivered were 50 Gy/25 fractions to the whole breast +/- regional nodes followed by a tumor boost of 21 Gy/3 fractions (equivalent dose delivered in 2 Gy= 86.9 Gy) by means of SABR or 20 Gy/8 fractions by means of IMRT (equivalent dose delivered in 2 Gy= 71.5 Gy). In this study, despite substantial heterogeneity in treatment delivery (some patients received also hyperthermia and concurrent chemotherapy), stage and biological subtype of disease included, with a median follow-up of 50 months, the 5-year local control was 93.4% (16)

The first level dose (40 Gy in 5 fractions, delivered every other day) was tested in the adjuvant setting and was deemed safe. Rahimi et al. in a phase 1 trial investigating a dose-escalated 5-fraction stereotactic body radiation therapy for partial-breast irradiation delivered in 75 patients after partial mastectomy reported only 1 DLT, which consisted of an acute grade 3 dermatitis in the intermediate dose level cohort. No DLT was observed at the highest dose of 40 Gy (13).

In this paper, we report the outcome of the first 10 patients treated to a total dose of 40 Gy in 5 fraction. All patients completed the treatment in 2 weeks without suspending the systemic treatment they were on, consisting of both hormone therapy and anti CDK4/6 inhibitors, capecitabine plus lapatinib and anti-HER2 therapy. The greatest toxicity was grade 2 moist desquamation occurring in 4 patients, which healed after appropriate treatment.

All patients with clinical symptoms experienced a clinical benefit, and were not exclude from the protocol as the SABR metastatic setting can have a palliative utility. In patients experiencing moist desquamation we recorded a medium maximum dose delivered to 10 cc of skin of 30.1 Gy. Similarly, Rahimi et al. showed that an average maximum dose to the skin of 38 Gy was related to the occurrence of radiation dermatitis (13).

Furthermore, the rate of response appears encouraging, even if the length of follow-up is still inadequate, especially for luminal patients. Also it can be influenced by the different biology of the disease included (HER2 + disease and luminal) as well as by the concomitant systemic therapy delivered.

After treatment of this first cohort of patients, no DLT occurred. Due to the high incidence of grade 2 skin toxicity in patients with tumors infiltrating the skin or located close to the skin (within 5 mm), we decided to expand this particular cohort to an additional 10 patients. The dose is being escalated in all other patients.

## Data availability statement

The raw data supporting the conclusions of this article will be made available by the authors, without undue reservation.

## Ethics statement

The studies involving human participants were reviewed and approved by Ethics committee Fondazione Campus Biomedico. The patients/participants provided their written informed consent to participate in this study.

## Author contributions

Conceptualization: SR. Data curation: SS, PM, and FP. Handle database: SC and VP. Formal analysis: RA and MM. Methodology: PO. Supervision: RD’A, VA, and GT. Writing: original draft EI. Writing: review and editing MF. All authors contributed to the article and approved the submitted version.
